# Gastrointestinal stromal tumour (GIST): British Sarcoma Group clinical practice guidelines

**DOI:** 10.1038/s41416-024-02672-0

**Published:** 2024-06-05

**Authors:** Ian Judson, Robin L. Jones, Newton A. C. S. Wong, Palma Dileo, Ramesh Bulusu, Myles Smith, Max Almond

**Affiliations:** 1 The Institute of Cancer Research, London, UK; 2 Royal Marsden NHS Foundation Trust, London, UK; 3 Southmead Hospital, Bristol, UK; 4 University College Hospital, London, UK; 5 Addenbrooke’s Hospital, Cambridge, UK; 6 Birmingham University Hospitals, Birmingham, UK

**Keywords:** Sarcoma, Oncology, Sarcoma

## Abstract

**Background:**

British Sarcoma Group guidelines for the management of GIST were initially informed by those published by the European Society of Clinical Oncology. This update was written by a group of experts to includes a discussion of the highlight improvements in our knowledge of the disease and recent treatment developments. The guidelines include sections on Incidence, Aetiology, Diagnosis, including risk assessment, Treatment and Follow-up.

**Methods:**

A careful review of the literature was performed to ensure that wherever possible recommendations are supported by the results of clinical trials or substantive retrospective reports. Areas of uncertainty are indicated appropriately.

**Conclusion:**

Guidelines represent a consensus view of current best clinical practice. Where appropriate, key recommendations are given and the levels of evidence and strength of recommendation gradings are those used by the European Society for Medical Oncology (ESMO).

## Incidence

Gastrointestinal stromal tumours (GISTs) are rare cancers, with an estimated unadjusted incidence of 1.5/100,000/year [1]. Data from the Rhȏne-Alpes region of France [2] and ‘NHS England Cancer Registry’ suggest an incidence of just under 11 per million per annum, equating to 650 clinically meaningful new cases a year in the UK, ~900 in total.

The median age at diagnosis is around 60–65 years, with a wide range. Occurrence in children, adolescents & younger patients is very rare, although paediatric GISTs represent a distinct subset, marked by female predominance, absence of *KIT*/platelet-derived growth factor alpha (*PDGFRA*) variants, gastric origin or multicentric location and possible lymph node metastases [3, 4]. The levels of evidence and strength of recommendation gradings used in these guidelines are adapted from a publication by the Infectious Disease Society of America [5]. Table 1 indicates the grading method used. Grades are included in brackets after key recommendations.

**Table 1:** Definition of ‘Levels of evidence’ and ‘Grades of recommendation’.

**Levels of evidence**
I Evidence from at least one large randomised, controlled trial of good methodological quality (low potential for a bias) or meta-analyses of well-conducted randomised trials without heterogeneity
II Small randomised trials or large randomised trials with a suspicion of bias (lower methodological quality) or meta-analyses of such trials or of trials with demonstrated heterogeneity
III Prospective cohort studies
IV Retrospective cohort studies or case-control studies
V Studies without control group, case reports and experts’ opinions
**Grades of recommendation**
A Strong evidence for efficacy with a substantial clinical benefit, strongly recommended
B Strong or moderate evidence for efficacy but with a limited clinical benefit, generally recommended
C Insufficient evidence for efficacy or benefit does not outweigh the risk or the disadvantages (adverse events, costs, …), optional
D Moderate evidence against efficacy or for adverse outcome, generally not recommended
E Strong evidence against efficacy or for adverse outcome, never recommended

## Aetiology

In the majority of cases the aetiology is unknown, although it is reported that patients with GIST are more likely to be diagnosed with another cancer than the general population [4, 6], suggesting a likely link with inherited increased susceptibility to cancer in some patients [7, 8]. In the majority of cases GIST is associated with an activating variant in either the *KIT* or *PDGFRA* gene. However, other rare variants may include *NF1 (*neurofibromatosis type 1, loss of function), *BRAF* (gain of function) or even more rarely, *RAS* genes (gain of function). Tumours lacking variants in *KIT* or *PDGFRA* were traditionally called ‘wild-type’ (WT). While tumours lacking variants in these genes but also *BRAF/RAS* and *NF1* have been dubbed ‘quadruple wild-type’ [9], it is more appropriate to specify those genes that have been tested, e.g. WT for *KIT, PDGFRA, NF1* and *BRAF/RAS*. Many such ‘wild-type’ tumours have a deficiency in succinate dehydrogenase (SDH), which may be due to a variant in one of the *SDH* genes (A,B,C or D), which can be either sporadic or inherited, or due to epigenetic gene silencing, in which case the gene affected is usually *SDHC*. Specific advice concerning the management of patients with paediatric and adolescent, ‘wild-type’ and syndromic GIST can be obtained via the web site www.pawsgistclinic.org.uk. Patients in this sub-group can apply to attend a PAWS-GIST clinic and contribute to research into more effective treatment for these diseases.

Several genetic syndromes are linked to GIST:

- The Carney triad syndrome, comprising gastric GIST, paraganglioma and pulmonary chondroma (these may occur at different ages) [10]. Most of these show succinate dehydrogenase C gene (*SDHC)* promoter hypermethylation.
- Carney-Stratakis syndrome, characterised by a dyad of GIST and paraganglioma, is marked by germ-line variants of one of the *SDH* genes A, B, C or D [11, 12],
- Type-1 neurofibromatosis, associated with loss of function of *NF1*, whether sporadic or inherited, and absence of variants in *KIT* or *PDGFRA*. These GISTs are often multicentric, predominantly located in the small bowel [13]. Patients with a germline variant in *NF1*, have a lifelong increased risk of GIST as well as malignant peripheral nerve sheath tumour (MPNST). Currently there is no specific systemic treatment recommendation for *NF1* mutant GIST.
- Familial GIST, i.e. families with a germ-line variant of *KIT* or *PDGFRA*, are extremely rare, presenting with multiple GISTs at an early age.

## Diagnosis

The most common symptoms of GIST include upper gastrointestinal bleeding and anaemia, whilst larger tumours may present with abdominal pain/discomfort and a palpable mass. Small bowel GISTs may remain silent for a long period before presenting with an acute event such as haemorrhage or rupture. Symptomatic rectal GISTs may present with pain, obstruction and bleeding; oesophageal and gastro-oesophageal junction GISTs with dysphagia. Some patients may have non-specific systemic symptoms e.g. weight loss, night sweats and fever. Lack of awareness of the presenting features may lead to delayed diagnosis of GIST in some patients. Small GISTs may be asymptomatic and are often diagnosed incidentally during investigation for other conditions.

Small asymptomatic submucosal lesions <2 cm in diameter in the upper gastrointestinal tract and small intestine may be kept under surveillance, e.g. by EUS on an annual basis and biopsied or excised if they continue to grow, or for patient preference. Large retrospective studies suggest that routine surveillance for such lesions is unnecessary. For larger lesions it is necessary to make a histological diagnosis. In gastric tumours this is most commonly by fine needle aspirate or core needle biopsy under endoscopic ultrasound (EUS) guidance. CT- or ultrasound-guided biopsy may also be considered for very large (>10 cm) tumours.

It is preferable to obtain a pre-operative diagnosis even in easily resectable tumours to exclude differential diagnoses including leiomyosarcoma, germ cell tumour, lymphoma, benign and malignant neurogenic tumours and desmoid tumours, as these pathologies may require different treatment strategies. When performed appropriately, preoperative biopsy appears safe with minimal side effects and without oncological compromise, and may influence management if predictive of response to systemic treatment [14] Excision biopsy may be undertaken in circumstances where biopsy is impossible, e.g. some small intestinal GISTs and in symptomatic lesions such as bleeding gastric tumours. If a suspected GIST is being resected however, it should be resected as if it is a cancer and by appropriately trained surgeons in or linked to a sarcoma specialist centre.

In larger, more complex tumours that may require a multi-visceral resection or other potentially morbid surgery such as a total gastrectomy, it is essential to make every effort to obtain a pre-operative diagnosis, either by EUS or by image-guided percutaneous biopsy since systemic treatment is likely to be given to downstage the patient and this will be dependent on the diagnosis. If the diagnosis is GIST, mutational analysis is mandatory to exclude imatinib-resistant disease.

There may be concern regarding biopsy of cystic masses but while EUS biopsy, if feasible, is preferable to minimise the risk of peritoneal contamination and seeding, transcutaneous biopsy appears to be safe and it is usually possible to target a solid, viable component of the tumour [14]. If a patient presents with obvious metastatic disease, then a biopsy of an easily accessible metastatic focus should be performed and a laparotomy/laparoscopy for diagnostic purposes is usually unnecessary.

Pathologically, the diagnosis of GIST relies on morphological assessment and immunohistochemistry (IHC), the diagnosis being supported by CD117 and/or DOG1 immunopositivity [15, 16]. About 5% of GISTs are CD117 immunonegative, about 5% of GISTs are DOG1 immunonegative and about 1% of GISTs are immunonegative for both [17, 18]. The mitotic count has prognostic value and is more accurate and reproducible when expressed as the number of mitoses in a total area of 5 mm^2^, which is therefore recommended. If there is diagnostic doubt, particularly in CD117 and/or DOG1 immunonegative suspected GIST, molecular analysis for typical variants in *KIT* or *PDGFRA* may help confirm the diagnosis.

Molecular analysis has predictive value for sensitivity to molecular-targeted therapy, and prognostic value. The inclusion of *KIT/PDGFRA* molecular analysis in the diagnostic work-up of all GISTs is highly recommended but as a minimum, such analysis should be performed on all the following specimens: resected moderate or high-risk GISTs at any site; resected GISTs showing tumour rupture; biopsies diagnostic of GIST prior to neoadjuvant or adjuvant therapy; specimens from patients with unresectable and/or metastatic GIST; GISTs which are suspected to be syndromic. Patients with GISTs which show clinicopathological features suggestive of SDH deficiency (i.e. paediatric or young female patient, gastric location, multinodular ± multifocal, epithelioid or mixed cell type histology, nodal metastases) should also have IHC for SDHB and if negative, testing for sporadic or germline SDH mutation/epigenetic loss. It is essential that molecular analysis is performed in centralised laboratories which are enroled in an external quality assurance programme, and which have expertise in GIST genomic analysis.

**Fig. 1:**
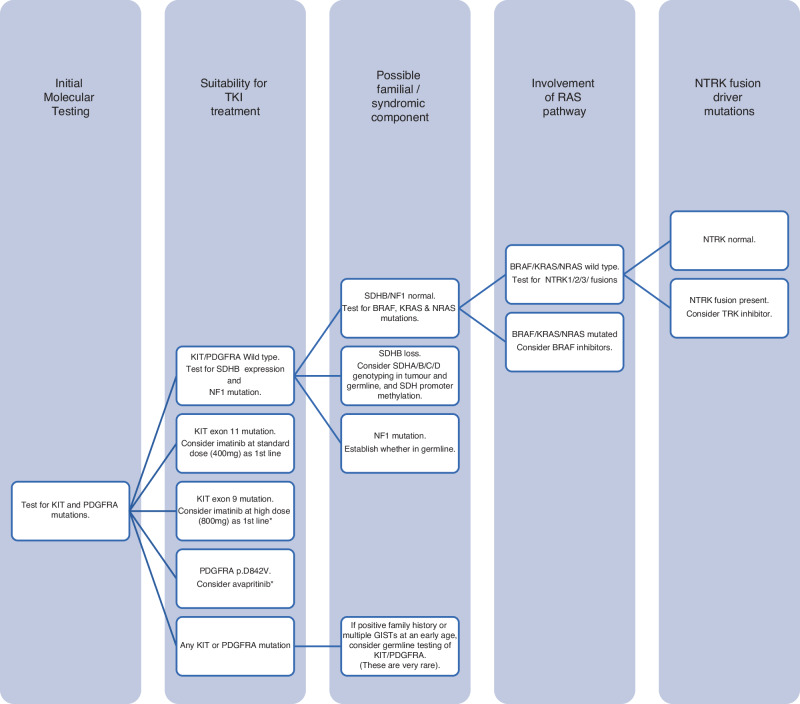
Algorithm indicating a sequential decision-making tool for molecular testing of newly diagnosed GIST. Note that imatinib 800 mg for KIT exon 9 mutant GIST and avapritinib for PDGFR exon 18 D842V mutant GIST are not approved by NICE.

For *KIT/PDGFRA* WT GISTs which are not NF1-related, IHC for succinate dehydrogenase B (SDHB) should be performed if available since loss of expression may assist the diagnosis and may help guide therapy. Further molecular analysis may demonstrate a loss of functional variant in a SDH gene or epigenetic loss, usually in *SDHC*. *KIT/PDGFRA* WT GISTs which do not show SDHB loss should then be analysed for *BRAF* and *RAS* variants; *BRAF* variants are especially important to exclude as they may be targeted therapeutically. Finally, GISTs which are WT for *KIT*, *PDGFRA* and *BRAF/RAS* and show no SDH-deficiency or NF1 association should be interrogated for *NTRK* fusions. GISTs with *NTRK* fusions are very rare but are potential candidates for TRK inhibitor therapy. Tumour molecular testing by Genomics England may identify other potential targets. For an idealised guide to the appropriate sequence of molecular analyses in relation to therapy see Fig. 1. Note, this may not accurately reflect local availability of molecular tests and neither ripretinib nor avapritinib are approved by NICE.

Collection of fresh/frozen tissue is encouraged, because new molecular pathology assessments can then be made at a later stage. Informed consent for tumour banking should be sought, so that the tissue is available for later analyses and research, provided that local ethical approval is in place. Consent forms for the National GIST Tissue Bank based at the Royal Marsden Hospital can be found at https://www.gistcancer.org.uk/national-gist-tissue-bank/.

**Key recommendations:**

1. Suspected GISTs larger than 2 cm in diameter should be investigated and a diagnosis made whenever possible. Often this can be done by EUS biopsy, provided the lesion is in the stomach, duodenum, or rectum. [IVC]
2. Small intestinal GISTs may not be accessible to EUS or percutaneous biopsy. If symptomatic, or at risk of causing imminent obstruction, these should be excised without a confirmed pre-operative diagnosis. Incidental small, i.e. <2 cm lesions do not require routine surveillance, alternatively a laparoscopic/robotic excision biopsy may be performed. [IVB]
3. The diagnosis of GIST should be established by a pathologist experienced in the disease and should include the use of IHC and, if necessary, molecular analysis (which should be performed by an accredited laboratory). [IVC]
4. If initial treatment with imatinib is planned, it is essential to confirm the diagnosis of GIST, since there is a wide differential [IVC]. It may be necessary to perform a percutaneous core needle biopsy if the tumour is inaccessible to EUS biopsy. Molecular analysis is particularly critical, since some GISTs are insensitive to tyrosine kinase inhibitors (e.g. the *PDGFRA* exon 18 D842V mutation). [IIB]

## Risk assessment for primary tumours with no evidence of metastatic disease

The TNM classification does not add to existing prognostic systems for GIST and its use is not recommended in this disease. Prognostic factors of proven value are the mitotic rate, tumour size and tumour site. Gastric GISTs have a better prognosis than small bowel or rectal GISTs. Tumour rupture through a serosal surface is an adverse prognostic factor and should be recorded, whether it took place before or during surgery. Molecular analysis data have not been incorporated into any risk classification so far, although some genotypes have a distinct natural history, e.g. *KIT/PDGFRA* WT GISTs tend to exhibit more indolent behaviour than *KIT* exon 11 variant disease.

A widely used risk classification was proposed by the Armed Forces Institute of Pathology (AFIP) [19], which distinguishes different risk levels compared with the 2002 National Institute of Health (NIH) Consensus criteria [15]. The key distinction is the difference in risk according to the site of the primary tumour, which is worse for a given size or mitotic index for sites other than the stomach and distinguishes between jejunal/ileal, duodenal and rectal primaries. A modified version of the NIH criteria also exists and has been employed for some previous trials (e.g. SSG XVIII/AIO) but the AFIP classification remains favoured for clinical use in the UK [20]. It is important to remember that mitotic index and tumour size are non-linear continuous variables, so that thresholds should be interpreted wisely. Prognostic contour maps have been generated via several series of GIST patients which incorporate the mitotic index and tumour size as continuous non-linear variables, while tumour rupture is considered in addition to tumour site; these contour maps have been validated against pooled data from 10 series and 2560 patients from the literature [21]. Standard risk stratification models may not predict risk of progression in SDH deficient GIST patients. In clinical practice we do not recommend using the AFIP or prognostic contour-based risk stratification in SDH deficient GISTs. A collaborative approach is needed to develop robust risk stratification models in this rare subtype of GIST [22]. Several nomograms and web or mobile phone applications are available to enable rapid risk category calculations to be made, which may assist multidisciplinary planning of patient management.

## Staging procedures

Staging procedures take into account the fact that most relapses affect the peritoneum and the liver. Contrast-enhanced abdominal and pelvic CT scan is the investigation of choice for staging and follow-up. Magnetic resonance imaging (MRI) or contrast-enhanced ultrasound may be alternatives, especially in younger patients where exposure to radiation should be limited. MRI provides better preoperative staging information for rectal GISTs. Chest CT scan or X-rays and routine laboratory testing complement the staging work-up of the asymptomatic patient but are not routinely required during follow-up since the incidence of pulmonary metastases is low. Evaluation of FDG uptake using an FDG-positron emission tomography (PET) scan, or FDG-PET–CT/MRI, can sometimes be useful, particularly when early assessment of response to tyrosine kinase inhibitor therapy is of special interest.

## Treatment

Treatment of GIST depends on the size and location of the tumour, the age and co-morbidity of the patient and the presence or absence of symptoms or complications such as perforation, bleeding or obstruction. Multidisciplinary treatment planning is essential. This should involve histopathologists, radiologists, surgeons and medical oncologists, as well as gastroenterologists and nuclear medicine specialists as applicable. Such teams are available in reference centres for sarcomas and GISTs, which treat a large number of GIST patients annually. Support staff, such as clinical nurse specialists, play a vital role and are not likely to be available, or have the appropriate expertise, outside specialised centres.

Small (<2 cm) incidentally detected tumours in the upper gastro-intestinal tract will either be very low risk, or entities whose clinical significance remains unclear. The standard approach to these tumours is EUS assessment, usually with fine needle aspiration or core needle biopsy to confirm the diagnosis, then annual follow-up, reserving excision for patients with enlarging tumours or those that become symptomatic. In some cases, patients may prefer definitive treatment (surgical excision) rather than surveillance. As indicated above, large retrospective studies suggest that routine surveillance for very low risk lesions is not indicated. Ultimately the management of small upper gastrointestinal GISTs is influenced by patient preference, age, life expectancy and comorbidities. Where surveillance is the preferred strategy, definitive evidence regarding the optimal radiological interval is lacking, but annual follow-up is considered reasonable, at least for a period. Patients with stable lesions for >3 years may be discharged. Follow-up is discussed in more detail below.

For a histologically proven GIST of 2 cm or greater, the standard treatment is excision in patients that are fit. In anatomically complex locations (e.g. at the gastro-oesophageal junction (GOJ) or second part of the duodenum (D2)) and in patients with significant comorbidities, a strategy of surveillance may be considered in tumours >2 cm depending on the wishes of the patient. In specialist centres, if expertise is available, one could consider a minimally invasive or robotic approach to resect these tumours in difficult to access anatomic areas, with the caveat that the published experience is limited to institutional series [23–26]. [IVC]

## Localised disease—surgery

Standard treatment of localised GIST is complete surgical excision of the lesion, with no dissection of clinically negative lymph nodes. Surgery should be performed by a subspecialty surgeon who is fully trained and experienced in radical anatomic site-specific cancer surgery linked to a specialist sarcoma centre, with the aim of achieving an R0 resection. When adjacent organs are involved, *en bloc* resection is recommended wherever possible. If laparoscopic or robotic excision is planned, the technique needs to follow the principles of oncological surgery [27].

Specific attention must be taken to avoid tumour rupture during resection as this is associated with a very high risk of relapse. This implies that for large, difficult to access, or cystic tumours, a minimally invasive approach may not be appropriate.

When R0 resection is considered difficult to achieve or is thought likely to result in major functional sequelae, e.g. total gastrectomy or abdominoperineal resection of rectum, neo-adjuvant imatinib should be considered provided there is a potentially sensitive mutation [28–31]. Neoadjuvant treatment may also be considered in patients with large tumours (>5 cm), or in challenging locations, with a molecular profile amenable to treatment with the aim of facilitating surgery and reducing the extent and need for multi-visceral resection.

Prior molecular analysis is crucial in order to prevent patients with less sensitive or resistant tumours (e.g. *PDGFRA* D842V mutations) from receiving therapy with imatinib, and also to allow appropriate dosing for patients with *KIT* exon 9 mutated tumours, i.e. 800 mg daily, if permitted, and potentially patients with *PDGFRA* D842V mutations which in the future may be pre-treated with avapritinib, or other new agents as they become available (see section on systemic treatment below). Early tumour response assessment is mandatory, so that surgery is not delayed in the case of non-responding disease. In the absence of molecular analysis, functional imaging, such as FDG PET-CT, makes it possible to assess the tumour response very rapidly, within a few weeks. There are limited data to guide the physician on when to stop imatinib before surgery, but it is usually stopped a week before surgery. However, much shorter intervals appear to be safe and it can be resumed promptly when the patient has recovered from surgery. If surgery follows treatment with sunitinib the interval needs to be longer to avoid problems with wound healing, owing to its inhibition of VEGFR and relatively long elimination half-life. Typically, surgery is performed after maximal tumour response, generally after 6–9 months of neo-adjuvant treatment.

The aim of surgery should be to resect the tumour with a disease-free margin, i.e. R0 resection. This is generally accepted to be a ≥1 mm margin but the meaning of precise margins is debatable in this disease and tumour at a diathermy margin is often considered acceptable since it is not routine to perform a repeat operation for marginal resection. In general, an organ sparing approach should be considered if feasible. In practical terms, as the majority of GISTs arise in the stomach, a wedge resection or similar organ sparing operation should be performed if possible, with the aim of achieving a ≥1 mm margin. There is no indication routinely to perform a lymph node dissection, with the possible exception of some SDH deficient GISTs, which may metastasise to nodes. It is reasonable to plan surgery based on the imaging findings at plateau, as opposed to on presentation. However, if an organ resection or multivisceral resection is required, it should be performed in a specialist centre, with an appropriately constituted surgical team with the expertise to safely resect the tumour and surrounding organs en bloc.

### Rectal GIST

The management of Rectal (or recto-vaginal space) GIST is complex, and a multidisciplinary approach is particularly helpful in these cases [30]. All suspected rectal GISTs should be biopsied and preferably excised after EUS assessment, regardless of tumour size. This is because GISTs at these sites have a higher risk of recurrence, and the local implications for surgery are more critical. Surgery should be considered for all cases, regardless of size. Great care needs to be taken in initial assessment, with dedicated rectal MRI, and an examination under anaesthesia may be considered. Surgical strategy needs to be tailored to the precise anatomic site and size of the tumour, in particular the relation to the sphincter complex. The approach should be carefully considered, as rectal GISTs may be accessed and resected via pararectal incisions, transanal approaches (including TAMIS), minimally invasive surgery including robotics and may need an abdominoperineal resection [30, 32–37]. When possible, an organ preserving approach should be considered. For larger tumours, a neoadjuvant approach should be considered, which may facilitate a more marginal excision, in particular when the tumour demonstrates response to imatinib [30, 38, 39]. In some cases, where surgery is not feasible, radiotherapy may be considered after, or in addition to, systemic therapy, to maintain local control. There is a relatively high rate of recurrence for rectal GIST post-surgery and marked regional variability in the surgical approach, which indicates a benefit for management of these cases in specialist centres [40, 41].

**Key recommendations**

1. Standard treatment of localised GIST is complete excision to achieve an R0 resection, i.e. ≥1 mm, if possible, with no dissection of clinically negative lymph nodes [IIIA].
2. Surgery should be performed by an appropriately trained cancer surgeon either in, or linked to, a specialist centre. This also applies to laparoscopic or robotic excision [IIIA].
3. If a total gastrectomy or abdominoperineal resection would be required to achieve an R0 resection consideration should be given to neoadjuvant therapy with imatinib, provided the tumour is not driven by a drug-resistant variant, e.g. *PDGFRA* exon 18 D842V mutation. A multidisciplinary approach is particularly important in the case of rectal GIST [IVA]

## Localised disease—adjuvant therapy

The risk of relapse following surgery can be substantial, as defined by available risk classifications. Adjuvant treatment with imatinib for 3 years was associated with improved relapse-free and overall survival compared with 1 year of therapy in a randomised trial in high-risk patients [42]. Previously, a placebo-controlled trial demonstrated that imatinib given for 1 year can prolong relapse-free survival in localised GISTs larger than 3 cm with a macroscopically complete resection [43]. Therefore, adjuvant therapy with imatinib for 3 years is standard treatment for patients with a high risk of relapse and a potentially sensitive driver variant, e.g. *KIT* exon 11. The use of adjuvant imatinib was approved by the National Institute for Care and Health Excellence (NICE) in their recent re-appraisal (https://www.nice.org.uk/guidance/ta326). This recommendation could be modified in the future once the results of the SSG XXII trial, which compared 3 years with 5 years of adjuvant imatinib treatment, are known. Patients with high risk of recurrence, particularly with intestinal and rectal tumours and sensitive mutations should be considered for adjuvant therapy. Marginal excision, i.e. an R1 resection, is not in itself an indication for adjuvant therapy if the tumour is otherwise not high risk.

Molecular analysis is critical to making a clinical decision regarding adjuvant therapy. Molecular analysis of tumours from the patients included in the SSG XVIII study which compared 1 versus 3 years of adjuvant imatinib showed that the largest benefit was observed in those patients with otherwise poor prognosis, i.e. those with *KIT* exon 11 deletions or insertion/deletions, (indels). The number of patients with other variants was small making it difficult to draw firm conclusions. However, patients with a good prognosis, e.g. those with *KIT* exon 11 substitutions, may still benefit from 3 years adjuvant imatinib [44, 45]. There is a consensus that *PDGFRA* D842V-mutant GISTs should not be treated with adjuvant therapy, given the lack of sensitivity of this genotype to imatinib. [Given the data supporting the use of a higher dose of imatinib (800 mg daily) in the presence of an exon 9 *KIT* variant in advanced GIST, clinicians might consider using this dose in the adjuvant setting for this genotype [46–48]. However, a retrospective analysis of patients treated with the 800 mg dose in the adjuvant setting did not show a benefit in terms of PFS or OS [49]. In addition, use of the larger dose is not supported by any controlled trial data in the adjuvant setting and it is not approved by NICE in the UK. There is consensus on avoiding adjuvant treatment in NF-1 related GISTs, which are insensitive to imatinib in the advanced setting. On the other hand, a consensus is lacking among experts about whether *KIT/PDGFRA* WT, SDH-deficient GIST should be treated with adjuvant therapy. This reflects their lower sensitivity to imatinib, as well as their peculiar natural history, which is often more indolent, but subgroup analyses of available randomised trials are too limited to provide sufficient evidence.

If there has been tumour rupture before or during surgery, there will have been spillage of tumour cells into the peritoneal cavity and therefore occult peritoneal disease can be assumed to exist. This puts the patient at a very high risk of peritoneal relapse. Therefore, these patients should be considered for adjuvant imatinib therapy. The optimal duration of treatment in these cases is unknown, given the uncertainty as to whether they should be viewed as having essentially metastatic disease, but should be at least 3 years, as for high risk resected GIST, and probably life-long.

**Key recommendations**

1. GIST should be managed by an experienced multidisciplinary team in a specialist centre. [IVB]
2. Pre-operative systemic therapy should be considered for those primaries where immediate resection is likely to be morbid, e.g. total gastrectomy, abdominoperineal resection or multi-visceral resection. In this situation mutational analysis is mandatory prior to the initiation of imatinib therapy. [IVB]
3. Patients at high risk of recurrence or distant relapse should receive 3 years of adjuvant imatinib, provided their tumour is not likely to be resistant to therapy
4. (i.e. particularly excluding tumours with a *PDGFRA* exon 18 D842V mutation). [IA]. Current risk stratification models are inaccurate in the case of SDH-deficient GIST and uncertainty remains concerning adjuvant therapy for this disease [IVB].

## Metastatic disease—systemic treatment

### Imatinib

In patients with inoperable and metastatic disease, imatinib is the standard treatment [50–52], including patients who had previously received the drug as adjuvant therapy without relapse during this treatment. This also applies to patients with metastases whose disease has been completely removed surgically, although surgery as a primary approach to metastatic GIST is not recommended.

The standard dose of imatinib is 400 mg daily. However, data have shown that patients with *KIT* exon 9 variants fare better in terms of progression-free survival (PFS) on a higher dose of 800 mg daily, which is therefore the standard treatment in this subgroup [53]. A report on the long-term follow-up of the European / Australasian clinical trial which compared 400 mg with 800 mg imatinib in patients with advanced GIST has shown a significant survival advantage for the initial use of the 800 mg dose in those with *KIT* exon 9 variants with a hazard ratio of 0.54 [54]. Treatment should be continued indefinitely, since treatment interruption is generally followed by relatively rapid tumour progression, even when lesions have been previously surgically excised [55]. At the start of treatment, the patient should be alerted to the importance of adherence to therapy, and of possible interactions with concomitant medications and foods, especially grapefruit, a potent inhibitor of the liver enzyme CYP3A4. They should also be given guidance about the best ways to handle any possible side effects. Dose intensity should be maintained by effective management of side effects, and a rational policy of dose reductions and interruptions should be applied if there is excessive, persistent toxicity. It has been reported that suboptimal plasma levels of imatinib are associated with a worse outcome, [56–58]. However, the use of a higher dose of imatinib in patients with progressive disease, even if low plasma levels can be demonstrated, is not currently approved by NICE.

Close monitoring of the tumour response should be carried out in the early phases of treatment. Follow-up should be continued throughout the treatment since the risk of secondary progression persists over time. Complete excision of oligometastatic or residual oligometastatic disease has been shown to be related to a good prognosis, provided the patient is responding to imatinib or sunitinib, but whether this is due to surgery or to patient selection [59–62] has never been demonstrated prospectively. Conducting a randomised trial did not prove feasible; thus, at the present time surgery may be discussed with the patient but not recommended on the basis of a definitive proof of benefit as regards improved progression-free survival. Surgical excision of progressive disease is not recommended, given the poor results in published series, but surgery or ablation of limited progression, such as the ‘nodule within a mass’, has been associated with a progression-free interval in the same range as for second-line treatment with sunitinib. So, this may be a palliative option in the individual patient with limited progression, while continuing imatinib. In selected cases, under the supervision of the MDT, surgery may be considered in patients responding to systemic therapy. The rationale and potential benefit of metastasectomy and cytoreduction need to be carefully balanced with the potential impact of surgery and likelihood of further progression.

Dose escalation of imatinib to 800 mg in the case of a GIST with a *KIT* exon 9 variant showing disease progression could be considered if the higher dose was not used initially, and if there have been changes in drug pharmacokinetics over time. The higher dose might also be useful for non-exon 9 variant GISTs in the case of some secondary molecular mutations [29]. However, in the UK this dose is not approved by NICE.

### Sunitinib

If there is confirmed progression, or rare intolerance to imatinib after all attempts to manage side effects have failed, the standard second-line treatment is the tyrosine kinase inhibitor (TKI) sunitinib [50]. This drug was proven to be effective in terms of PFS using a regimen of 50 mg daily 4 weeks on/2 weeks off. Data have been provided that continuous treatment with a lower daily dose of 37.5 mg is also effective and well tolerated, although no formal comparison has been performed within a randomised clinical trial. This schedule can therefore be considered an alternative on an individualised basis [63]. Flexibility in terms of dose and schedule is required in order to manage the side effects, such as diarrhoea and skin toxicity, which maintaining disease control. Not all patients resistant to imatinib respond to sunitinib particularly those with secondary variants affecting the activation loop domain of *KIT* and the *PDGFRA* D842V mutation.

### Regorafenib

Regorafenib is regarded as standard therapy for the third-line treatment of patients progressing on imatinib and sunitinib. A prospective placebo-controlled randomised trial demonstrated that regorafenib, at a dose of 160 mg daily on a 3 weeks on/1 week off schedule, significantly prolonged PFS in patients progressing after both imatinib and sunitinib [64]. The key distinction between sunitinib and regorafenib, as also previously shown with the analogue sorafenib, is its ability to inhibit tumours with secondary variants in the activation loop of *KIT*, especially in exon 17 [65, 66]. These variants are known to confer resistance both to imatinib and sunitinib, hence the value of regorafenib in this setting. As with sunitinib, flexibility in terms of dose and schedule is required in order to manage side effects and optimise disease control.

### Ripretinib

Ripretinib is a ‘switch kinase’ inhibitor, which acts allosterically by altering the shape of the KIT molecule, specifically by inhibiting movement of the activation loop, rather than by binding at the active ATP-binding site. It was studied in a randomised, placebo-controlled trial in patients with advanced GIST who had progressed after treatment with 3 or more TKIs. The study showed a significant improvement in progression-free survival (PFS) compared with placebo (HR 0.15; 95% CI: 0.09, 0.25; *p* < 0.0001) [67]. The median PFS on ripretinib was 6.3 months (95% CI: 4.6, 6.9) compared with 1.0 month (95% CI: 0.9, 1.7) on placebo. The median OS ripretinib arm was 15.1 months (95% CI: 12.3, 15.1) compared with 6.6 months (95% CI: 4.1, 11.6) on placebo (HR 0.36, 95% CI: 0.21, 0.62). Side effects included fatigue, lipase increase, hypertension and electrolyte disturbances. A clinical trial comparing ripretinib with sunitinib after treatment with imatinib failed to show an improvement in PFS overall, although it had a more favourable side effect profile, including patient reported outcomes [68]. In the *KIT* exon 11 subgroup objective response rate was superior with ripretinib (23.9%) compared with sunitinib (14.6%) and similarly median PFS in this group was longer 13.3 months for ripretinib and 10.8 months for sunitinib. This difference was not statistically significant. In the *KIT* exon 9 group PFS was greater on sunitinib. Ripretinib was approved by the FDA in May 2020 and by the EMA in 2021 as 4th line treatment. Ripretinib is not currently approved by NICE.

### Avapritinib

Avapritinib was developed specifically for patients with GIST harbouring the *PDGFRA* exon 18 D842V mutation. It was first studied in a single-arm phase I study in GIST patients with *PDGFRA* exon 18 variants (and *KIT* variants), including 38/43 with *PDGFRA* D842V mutations. The trial concluded that a dose of 300 mg daily was safe and tolerable. In patients with *PDGFRA* exon 18 D842V mutations there were 49 of 56 responses, with 44 partial responses (79%) and five complete responses (9%) [69]. Reported side effects have included neurocognitive disturbances, nausea, fatigue, diarrhoea, oedema and skin rash. Avapritinib was approved by the FDA specifically for GIST patients with the *PDGFRA* exon 18 D842V mutation in January 2020. Analysis of the dose escalation and expansion cohorts of the Phase 1 avapritinib trial in patients with advanced GIST after three or more lines of prior therapy showed activity with a response rate of 17%, median duration of response of 10.2 months and median PFS of 3.7 months [70]. However, a randomised phase III study comparing avapritinib with regorafenib in patients pre-treated with imatinib and sunitinib, predominantly in patients with *KIT-*mutant tumours, did not show a significant difference in median PFS between the two arms [71]. Avapritinib remains the most active treatment for *PDGFRA* D842V mutant GIST. This agent has not been approved by NICE and currently doctors treating *PDGFRA* D842V mutant GIST patients will need to complete an NHS individual funding request to apply for access to prescribe avapritinib.

If a TKI is well tolerated, and if it is perceived that the patient is continuing to derive clinical benefit from the treatment, some clinicians continue TKI treatment beyond RECIST progression on the basis that this may slow progression and maintain quality of life. There may be theoretical reasons to support this view, given that not all cancer cells in a tumour will have developed resistance to therapy. However, there is no prospective clinical evidence to support this practice and no recommendation can be given in support of this practice in these guidelines. It is important that all patients with advanced disease be considered for participation in clinical trials when available in order to maximise recruitment in this uncommon disease and help improve outcomes.

## Metastatic disease—non-surgical local therapy

Selected patients with limited liver metastatic disease may be amenable to surgery or non-invasive techniques such as radiofrequency ablation (RFA) or stereotactic body radiotherapy (SBRT) after maximum response to imatinib, if there is evidence of localised disease progression, or in the setting of a symptomatic lesion, or potentially symptomatic lesion where there remains the potential for further lines of systemic therapy. The use of RFA is restricted to tumours in the region of 3 cm in maximum diameter and is less likely to be a suitable approach for lesions adjacent to large vessels or superficial lesions, especially if displacing the liver capsule. However, larger isolated lesions and superficial lesions may still be suitable for surgical resection, either by partial hepatectomy or wedge resection. Dedicated liver MRI scans and when appropriate CT-PET scans may be required to determine whether this is a legitimate approach by excluding other occult active disease.

Radiotherapy may be a useful local therapy in GIST under certain circumstances in the advanced disease setting. If there is a single site of disease that is progressing on a TKI and can be encompassed within a radiotherapy treatment field, radiotherapy delivered to a moderate or high dose can offer local tumour control and possibly prolong the use of the TKI [72]. Radiotherapy, including Selective Internal Radiation Therapy, can be used for metastatic liver disease and external beam radiotherapy at lower doses can be used to palliate symptomatic disease, causing pain or bleeding, for example. [IVB]

## Response assessment

Response assessment is complex and early progression should be confirmed by a team experienced in treating GIST. Anti-tumour activity translates into tumour shrinkage in most patients, but some patients may show only changes in tumour ‘density’ on imaging, these changes sometimes precede a reduction in tumour volume. Such changes in tumour radiological appearance should be considered as indicative of tumour response. Tumour size may even increase in the short term but if tumour density on CT scan is decreased this may still indicate tumour response [73, 74]. Even the apparent ‘appearance’ of new lesions may be due to them becoming less dense, or cystic, especially in the liver. Therefore, both tumour size and tumour density on CT scan, or consistent changes on MRI or contrast-enhanced ultrasound, should be considered when determining tumour response. FDG PET-CT has proved useful in the early assessment of tumour response, when prediction of response is valuable, for example in the case of preoperative therapy, or when response is in doubt. However, a small proportion of GISTs have no FDG uptake. The absence of tumour progression at 6 months [75] is also equivalent to a tumour response. Conversely, tumour progression may not always be accompanied by changes in tumour size. For example, an increase in the tumour density shown by contrast enhancement within a previously responding low density tumour lesion, may be indicative of tumour progression. A typical progression pattern is the ‘nodule within the mass’, in which a portion of a responding lesion becomes hyper-dense [76].

**Key recommendations**

1. Imatinib is the treatment of choice for patients with unresectable or metastatic disease and is given until progression at the standard dose of 400 mg daily [IA]. Data suggest that there is a benefit for a larger dose for those patients whose tumours have an exon 9 variant in *KIT*, though this is not currently recommended by NICE [IIA]. Imatinib is not recommended for patients with *PDGFRA* exon 18 D842V mutant disease [IC].
2. Isolated progression may be amenable to surgery or other local measures, such as radiofrequency ablation [IVB].
3. Standard second line treatment is sunitinib, which may be given at the recommended dose of 50 mg daily for 4 weeks every 6 weeks, or 37.5 mg daily continuously [IA].
4. Standard 3^rd^ line treatment is regorafenib [IA].
5. The most effective treatment currently available for *PDGFRA* D842V-mutant GIST is avapritinib. It is not currently approved by NICE [IIB].
6. Ripretinib appears to be a useful drug for later lines of therapy. However, a randomised study comparing it with sunitinib failed to show an improvement in PFS. It has not been approved by NICE [IA].

## Follow-up

There are no published data to suggest what the optimal follow-up policy should be for surgically treated patients with localised disease. Relapses occur most often in the liver and/or the peritoneal cavity. Other sites of metastases, including bone and brain are uncommon, but may be less unusual during the course of metastatic disease following prolonged treatment with several lines of therapy. The mitotic rate may affect the rate at which relapses occur. Risk assessment based on the mitotic count, tumour size and tumour site may be useful in choosing the follow-up policy for an individual patient. High-risk patients generally relapse within 1–3 years from the end of adjuvant therapy. Low-risk patients may relapse later, given that the disease is likely to be slower growing. Routine follow-up schedules differ across institutions which reflects the fact that optimal follow-up schedules are not known. In some centres, high-risk patients who have undergone resection of their primary undergo a routine follow-up abdominal CT or MRI scan every 3–6 months during adjuvant therapy, for 3 years. This high frequency of surveillance is because of the need to manage the side effects of the therapy. On cessation of adjuvant therapy follow up is every 3 months for 2 years, then every 6 months for another 3 years, after which follow up is annual for another 5 years. Patients with high-risk tumours not given adjuvant therapy, for whatever reason, have generally been followed up 3 monthly for 2 years, 6 monthly for 3 years and then annually for a further 5 years. Radiation exposure is a factor to consider when choosing the imaging modality for long term follow-up. Abdominal MRI is an acceptable alternative to CT which can be used, especially in younger patients, to avoid the risks associated with frequent radiation. It must be acknowledged that the benefit of intensive follow-up is not based on strong evidence. However, localised recurrences, if resected or ablated can be treated with curative intent and the bulk of recurrent disseminated disease at the time this is detected may have a survival impact. Research is clearly required to optimise follow-up regimens.

For intermediate and low-risk tumours, the usefulness of routine follow-up is not known. If follow-up is performed, it will usually be an abdominal CT or MRI scan, or ultrasound, every 6–12 months for 5 years, then annually up to 10 years, as per ESMO guidelines. As previously discussed, an analysis of pooled population GIST patient cohorts was performed by Joensuu and colleagues intended to clarify who might benefit from adjuvant therapy [21]. They included 2560 patients in the study and found that using modified NIH criteria patients with intermediate risk, excluding those with tumour rupture, had a similar prognosis to those with low risk and do not need adjuvant treatment. High risk patients had a distinctly worse rate of relapse. Risk stratification methods that address the continuous and non-linear nature of criteria such as tumour size and mitotic count, and take tumour rupture into account, such as the contour maps described in the paper, were more accurate in predicting recurrence but did not change the overall conclusion regarding the decision about adjuvant treatment. Recent reports also cast doubt on the value of routine follow-up for patients with low risk disease. For example, d’Ambrosio et al. followed up 790 patients with low risk disease according to AFIP criteria. Patients with tumour rupture, very small, i.e. <2 cm diameter tumours or those having had neo-adjuvant therapy were excluded. Patients had an abdomino-pelvic CT over 6 months for 5 years, then annually up to 10 years. 42 patients relapsed, 33 of whom developed metastases. Nine patients relapsed after 10 years. Relapses were mainly detected in the first 2 years after surgery. With a median follow-up of nearly 6 years 5-year DFS was 95.5%, GIST specific DFS was 99.8%; OS 96.1%, 10 DFS was 96.1%; OS 91% [77]. Patients with *KIT-*mutant tumours had a worse prognosis, those with gastric primaries did better. They proposed that patients with gastric primaries and no symptoms at presentation be followed every 6 months with USS every 6–12 months. Those with non-gastric primaries and *KIT* mutations should have CT every 6 months for 2 years then annually, or alternating with USS. In both groups MRI could be discussed as an option to limit radiation exposure.

Another large retrospective series of 649 patients from three Scandinavian centres looking at outcomes in very low, low and intermediate risk GIST patients. Risk stratification used both modified NIH and AFIP criteria. At a median follow-up time of 50.5 months there were 8 relapses, 7 in the low-risk group, 1 in the intermediate risk group. The 5 years relapse-free survival rate for the whole group was 99.1% being 100, 98.5 and 100% for the intermediate, low and very low risk groups respectively. The authors of this study concluded that routine follow-up was not beneficial for non-high risk patients [78].

Very low-risk GISTs do not require routine follow-up, and may be discharged after a normal CT although one must be aware that the risk of progression is not zero.

**Key recommendations**

1. Patients with high risk disease on adjuvant therapy should be followed up with cross-sectional imaging every 3–6 months during their first 3 years of treatment, 3 monthly for 2 years following cessation of treatment and thereafter every 6 months for 3 years and then annually for at least 5 years. [IVC]
2. Patients with high risk disease not receiving adjuvant treatment should follow the post-adjuvant surveillance scheme. [IVC]
3. For intermediate risk patients, according to AFIP criteria, 6 monthly scans for 5 years followed by annual scans is considered reasonable, but may be unnecessary according to some recent reports. The optimum duration of follow-up is unknown. [IVC]
4. In low risk GIST the role of routine surveillance imaging is unclear. Annual CT or USS for 5 years may be considered and clinical follow-up to check for a second malignancy is sensible, given the high frequency of second tumours in this disease. [IVC]
5. Very low risk patients do not require routine surveillance. [IVC]
